# Microtubule destabilization is a critical checkpoint of chemotaxis and transendothelial migration in melanoma cells but not in T cells

**DOI:** 10.1080/19336918.2021.1934958

**Published:** 2021-06-21

**Authors:** Francesco Roncato, Ofer Regev, Sandeep Kumar Yadav, Ronen Alon

**Affiliations:** Department of Immunology, Weizmann Institute of Science, Rehovot, Israel

**Keywords:** Cytoskeleton, motility, cancer, metastasis, taxol

## Abstract

Microtubules (MTs) control cell shape and intracellular cargo transport. The role of MT turnover in the migration of slow-moving cells through endothelial barriers remains unclear. To irreversibly interfere with MT disassembly, we have used the MT-stabilizing agent zampanolide (ZMP) in Β16F10 melanoma as amodel of slow-moving cells. ZMP-treated B16 cells failed to follow chemotactic gradients across rigid confinements and could not generate stable sub-endothelial pseudopodia under endothelial monolayers. In vivo, ZMP-treated Β16 cells failed to extravasate though lung capillaries. In contrast to melanoma cells, the chemotaxis and transendothelial migration of ZMP-treated Tcells were largely conserved. This is afirst demonstration that MT disassembly is akey checkpoint in the directional migration of cancer cells but not of lymphocytes.

## Introduction

The actomyosin cytoskeleton is key for all types of cell motility [1]. Microtubules (MTs) are additional essential components of the eukaryotic cytoskeleton. MTs are highly dynamic polar polymers expressed by all eukaryotic cells [2]. These cylindrical hollow polymers consist of αβ-tubulin heterodimers that arrange longitudinally head to tail to form protofilaments [3,4]. They play a pivotal role in vital cell functions, including cell division, morphogenesis, and intracellular transport [5]. MTs regulate chromosome movements during mitosis, organelle positioning, intracellular transport of membranous vesicles, and signal transduction [6,7]. MT contributions to directional cell migration and chemotaxis, as well as to nuclear squeezing through mechanical barriers, are still unclear as they vary with both the cell type, cue, and barrier [2,8].

Both leukocytes and tumor cells share F-actin-rich protrusions at the leading edge and actomyosin contraction at the uropod [9–12]. The organization and functions of MTs in these compartments during cell migration differs, however, fundamentally, between slow-moving mesenchymal cells and fast-moving cells like leukocytes. In the former (e.g., fibroblasts or tumor cells), MTs play key roles in the mobilization of vesicles essential for the growth and stability of lammelipodia [1,13], whereas in the latter, ameboid-like fast-moving leukocytes like T cells and neutrophils, MTs are enriched behind the nucleus and are thought to regulate contractility [14]. The contribution of MTs to compartmentalized Rho and myosin signaling at the back of polarized cells have been mainly studied by the disruption of MTs [15–17]. Nevertheless, the effects of interference with MT disassembly on directional cell migration are much less understood. In both types of cells, MT disassembly releases sequestered Rho GEFs that activate the Rho GTPase and its downstream myosin effectors at the uropod [15,18–20]. In slow-moving cells, however, MT assembly also contributes to the stability of the leading edge by providing a scaffold for signaling components [21,22]. A similar contribution of MT assembly to directed motility of fast-moving cells like leukocytes has not been demonstrated.

MT dynamics is endogenously regulated by MT-associated proteins (MAPs) and various deacetylases including histone deacetylases (HDACs) [23,24]. MAPs include motor proteins such as kinesins and dyneins, plus-end tracking proteins such as EΒ3, centrosome-associated proteins, and enzymatically active and structural MAPs [25–27]. HDACs are recruited by specific MT assemblies that are heavily acetylated, and by removing their acetyl moieties, facilitate their dissociation and thereby cell migration [28]. Dissecting the spatial and temporal contribution of MT turnover to specific steps of cell migration by genetic manipulation of MAPs is limited due to the critical function of MTs in mitosis [2,29]. Specific MT-stabilizing agents (MSAs) have therefore been useful in dissecting the functions of MTs in various modes of cell migration [30]. Taxol derivatives are the most widely used MSAs [29,31]. In addition to their extensive use as mitogenic inhibitors in various chemotherapies [32], these derivatives can interfere with specific cell motility machineries. The inhibitory mechanisms of taxol derivatives on cell motility vary not only with the cell type but also with the adhesive and chemotactic cues as well as the type of mechanical barrier the taxol-treated cells encounter [33,34]. For instance, both directional and random migration of HUVEC stimulated by multiple angiogenic factors is inhibited by taxol derivatives via interference with the MT-organizing center (MTOC) repositioning at the leading edge [35] and prolonging the turnover of focal adhesions [36].

The role of MT stability in tumor cell crossing of cellular barriers is unclear, whereas the role of MTs in directed migration of leukocytes is better understood [10,11]. In the present study, we have compared the contribution of MT dynamics to directional cancer cell and immune cell migration through confined spaces as well as through endothelial barriers, a motility known as transendothelial migration (TEM), that is a critical checkpoint in cancer metastasis [37,38]. We chose the prototypic metastatic Β16F10 melanoma as a model for cancer cell TEM and metastasis due to its ability to cross murine endothelial monolayers in vitro and extravasate the pulmonary capillary bed in vivo [39]. We also chose effector T cells as a prototype of highly migratory leukocytes that cross endothelial monolayers in response to chemotactic cues [40].

Zampanolide (ZMP) is a novel MSA, inhibitor of MT disassembly that irreversibly binds MTs at the same β-tubulin site occupied by taxol and thereby stabilizes these bundles and interferes with normal MT remodeling [41]. In wound scratch assays, ZMP treatment inhibited migration of human umbilical vein endothelial cells (HUVECs) and fibroblasts [42]. We chose this drug because its irreversible inhibitory properties have enabled us to pretreat our Β16F10 cells before allowing these cancer cells to interact with endothelial cell (EC) monolayers, which had not been co-exposed to the drug. Our results suggest that MT destabilization in Β16 melanoma cells is instrumental for the directional motility of these cells through rigid barriers, i.e., transwell filters, toward both soluble and immobilized attractants, as well as for the completion of TEM both in vitro and in vivo. During TEM, intact MT destabilization appears critical for the ability of the tumor cell to generate normal pseudopodia and translocate its nucleus into this large cell protrusion, a critical step in TEM. On the other hand, interference with MT turnover in T cells does not inhibit their ability to generate normal pseudopodia and translocate their nuclei into these protrusions during chemotaxis and TEM. We propose that MT disassembly is a critical checkpoint for directional migration of melanoma cells but is dispensable for directional lymphocyte migration.

## Materials and methods

### Cells

Murine melanoma cells (Β16F10, ATCC CRL-6475, gift from Prof. Lea Eisenbach, Weizmann Institute of Science) were grown in Dulbecco's Modified Eagle medium (DMEM) supplemented with 10% fetal bovine serum (FBS). Murine brain ECs (bEnd.3, ATCC CRL-2299, gift from Prof. Britta Engelhardt, Theodor Kocher Institute, University of Bern) were cultured in DMEM medium supplemented with 10% FBS and 2 mM L-glutamine. human dermal blood endothelial cells (HDBECs, PromoCell, Cat# C-12211) were grown in EC growth medium (PromoCell, Cat# C-22020) according to the supplier’s instructions and were used at passages 2–3. All the media were supplemented with 1% penicillin–streptomycin–amphotericin B solution. All cell cultures were maintained at 37°C in a humidified incubator in the presence of 5% CO_2_. Human T cells were isolated from citrate-anti-coagulated whole blood of healthy donors by dextran sedimentation and density separation over Ficoll–Paque™ Plus (GE Healthcare, Cat# 17-1440-03) as described [43]. For generation of T effectors, isolated lymphocytes were seeded on plates coated with anti-CD3 (BioLegend, Cat# 300314, RRID:AB_314050) and anti-CD28 (BioLegend, Cat# 302914, RRID:AB_314316) monoclonal antibodies (mAbs) for 48 h in T cell medium (RPMI-1640 supplemented with 10% FBS, 1 mM sodium pyruvate, 2 mM L-glutamine, and 50 µM β-mercaptoethanol), and then cultured for 9–12 d in the presence of IL-2 (PeproTech, Cat# 200-02) as previously described [44]. A day before the experiment, effector lymphocytes were washed and incubated overnight in fresh IL-2-containing T cell medium. All in vitro experiments with human leukocytes were approved by the Institutional Review Board of the Weizmann Institute of Science.

### Mice

Wild-type mice (WT) on C57BL/6 background were maintained in a pathogen-free facility, and all animal procedures were approved by the Animal Care and Use Committee of the Weizmann Institute of Science. Male 7 to 8-week-old mice were used in all experiments.

### Imaging and analysis of melanoma TEM

The transmigration assay of tumor cells was performed under shear-free conditions. Murine endothelial bEnd.3 cells (8 × 10^4^) were seeded in a μ-Slide VI0.4 ibiTreat (ibidi), precoated with gelatin (Sigma-Aldrich, Cat# G9391), 1% in double-distilled water (DDW) for 30 min at 37°C. A day later, Β16F10 cells were pretreated with 1 µM jasplakinolide (Millipore, Cat# 420107) for 30 min, 50 nM ZMP solubilized in dimethyl sulfoxide (DMSO), or carrier control (DMSO, 1:2000 dilution) for 3 h. Synthetic ZMP (gift from José Fernando Díaz, Centro de Investigaciones Biológicas, Madrid, Spain), was used at a concentration of 50 nM as described previously [41] and validated to be nontoxic for the B16F10 cell line for a period of at least 8 h. Cells were subsequently labeled with 20 µM Hoechst 33342 (Thermo Fisher Scientific, Cat# 62249), for 5 min at 37°C, resuspended in binding medium (Hank’s balanced-salt solution 1X containing 2 mg/ml bovine serum albumin (BSA) and 10 mM HEPES, pH 7.4, supplemented with 1 mM CaCl_2_ and 1 mM MgCl_2_), and introduced in the ibidi chamber over a confluent bEnd.3 monolayer. Images were acquired at a rate of one frame every 4–5 min for 4 h using an IX83 inverted microscope (Olympus) equipped with UPlanFLN 20×/0.50 Ph1 ∞/0.17/FN 26.5 objective (Olympus), 49,000-ET-DAPI filter set (Chroma). ORCA-Flash4.0LT camera, model: C11440-42 U (Hamamatsu). Temperature was maintained at 37°C throughout the assay. For analysis of migratory categories, tumor cells in different fields of view (10–15 cells per field) were individually tracked and categorized using cellSense software 1.16 (Olympus). Close monitoring of individual frames allowed the discrimination of transmigrating tumor cells (TEM) from tumor cells that failed to complete TEM either because of inability to protrude through endothelial junctions (SA) or squeeze their nuclei through these junctions and underneath the endothelial monolayer (SEP). Notably, we did not observe intercalation of individual tumor cells in between ECs [45]. Fiji software (https://fiji.sc), was used to manually outline the cell’s leading edge and nucleus, and to incorporate time codes, labels, and scale bars into video segments. Illustrations (Figure 2a,e) were created with Adobe Illustrator CS6.

### Imaging of MT dynamics

Β16F10 cells (1.5 × 10^5^) were transiently transfected with mCherry-alpha-tubulin encoding plasmid (Addgene #49149) using Lipofectamine® 2000 (Thermo Fisher Scientific, Cat# 11668), according to the manufacturer’s instructions. Then, 24 h post transfection, the cells were treated with 50 nM ZMP or carrier control (DMSO 1:2000 dilution) for 3 h. Cells were washed twice with warm PBS, removed from the culture plate by trypsinization, labeled with 20 µM Hoechst 33342, resuspended in binding medium (composition described above), and introduced into a μ-Slide VI0.4 ibiTreat (ibidi) over a bEnd.3-deposited basement membrane extracellular matrix (ECM) (isolated by removal of cultured bEnd.3 cells with 10 mM EDTA 30 min earlier). Tumor cell images were acquired at a rate of one frame every 4–5 min for 1 h using an IX83 Inverted Microscope (Olympus) equipped with PLAPON 60×OPh/1.4 objective (Olympus) with a Chroma filter set. ORCA-Flash4.0LT camera, model: C11440–42 U (Hamamatsu). Temperature was kept at 37°C throughout the duration of the assay. Background was subtracted for both the fluorescent channels, and 2D-deconvolution was performed in the mCherry channel using cellSense software 1.16 (Olympus). FiJi (SciJava) software was used for title and time code labeling.

### Acetylated tubulin immunofluorescence

Β16F10 (4 × 10^4^) cells pretreated with 50 nM ZMP or carrier control (DMSO 1:2000 dilution) for 3 h were trypsinized and seeded into a μ-Slide VI0.4 ibiTreat (ibidi) coated with fibronectin (5 µg/ml at 37°C for 45 min). Human Effector T cells (at a density of 2 × 10^6^/ml) pretreated with 100 nM ZMP (a nontoxic dose found optimal for MT stabilization) or carrier control (DMSO 1:1000 dilution) for 6 h were washed twice with warm PBS, settled into a μ-Slide VI0.4 ibiTreat (ibidi) precoated with 100 ng/ml CXCL12 (PeproTech, Cat# 300-28A) overnight, and 0.01% poly-L-lysine (Sigma-Aldrich, Cat# P4707) for 1 h at room temperature (RT). After 30 min, cells were fixed with paraformaldehyde (4% in PBS) for 15 min at RT and permeabilized with a solution of 0.25% Triton X-100 for 15 min at RT. Cells were then incubated with anti-acetylated α-tubulin antibody (Santa Cruz Biotechnology Cat# sc-23950, RRID:AB_628409) for 1 h at RT in a solution of 0.025% Triton X-100 and 1% BSA in PBS. Cells were washed three times with PBS and labeled with a Alexa Fluor488-conjugated secondary antibody (Thermo Fisher Scientific, Cat# A-21202, RRID:AB_141607), for 1 h at RT, and further labeled with 20 µM Hoechst 33342 for 5 min at RT. Cells were imaged using an IX83 inverted microscope (described above) equipped with a PLAPON 60×OPh/1.4 objective (Olympus) using a Chroma filter set.

### Transwell migration assay for tumor cells and effector T cells

**Figure 1.**
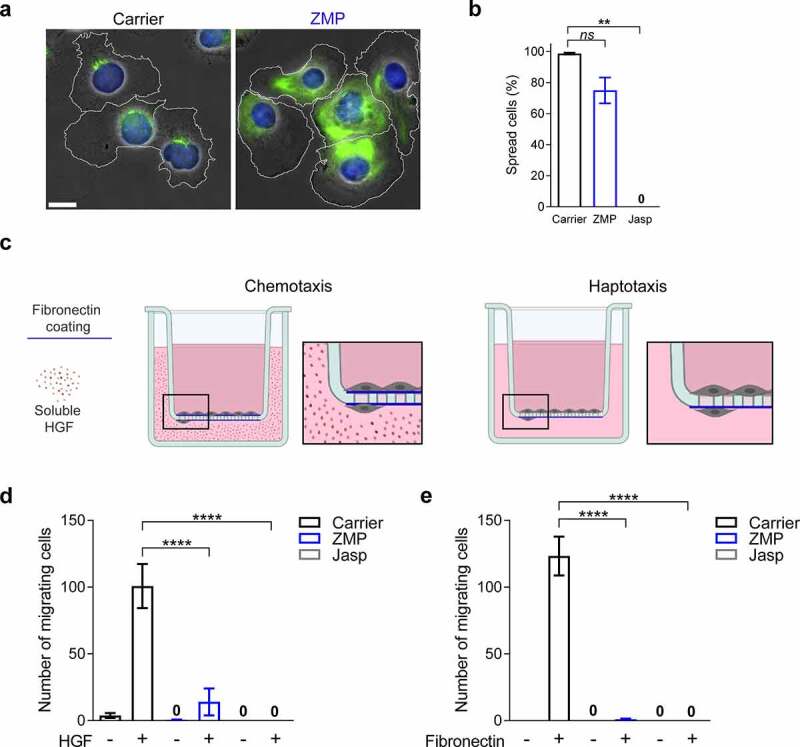
Stabilization of acetylated α-tubulin abolishes chemotaxis and haptotaxis of B16F10 melanoma cells

Fibronectin (1.5 µg/ml in PBS) was coated for 30 min at 37°C onto both sides (for tumor chemotaxis assays) or only on the bottom side (for tumor haptotaxis assays) of 8 µm hanging cell culture inserts (Millipore, MCEP24H48). Β16F10 cells pretreated with 1 µM jasplakinolide for 30 min, 50 nM ZMP, or carrier control (DMSO 1:2000 dilution) for 3 h were resuspended in DMEM containing 0.1% BSA and introduced (4 × 10^4^) into the top chamber. DMEM with 0.1% BSA was inserted in the lower chamber in the presence or absence of hepatocyte growth factor (HGF, PeproTech, Cat# 315-23) or other chemoattractants: vascular endothelial growth factor A(VEGF-A, PeproTech, Cat# 450-32), epidermal growth factor (EGF, PeproTech, Cat# 315-09), C-X-C motif chemokine ligand 12 (CXCL12, PeproTech, Cat# 250-20A), and slit guidance ligand 2 (SLIT2, R&D Systems, Cat# 5444-SL). After 4 h at 37°C with CO_2_, cells were fixed with paraformaldehyde (4% in PBS) for 15 min and stained with crystal violet (3% in DDW) for additional 15 min, both at RT. Cells on the upper side of the filter were scraped using a cotton swab, whereas cells located on the bottom side were imaged using a SZX16 stereo microscope (Olympus) equipped with SDF PLAPO 1XPF objective (Olympus) set at 10× magnification. Human effector T cells (at a density of 2 × 10^6^/ml) were pretreated with 100 nM ZMP or carrier control (DMSO, 1:1000 dilution) for 6 h, washed twice, and resuspended in binding medium (as described above). 2 × 10^5^ cells were seeded in the upper chamber of a 6.5 mm transwells with 5 or 3 µm pore uncoated polycarbonate membrane inserts (Corning, Cat# 3421 and Cat# 3415). The bottom chambers were filled with binding medium alone or supplemented with CXCL12 at 100 ng/ml. T cells were allowed to migrate across the filters at 37°C for 30 min. Subsequently, the transwell inserts were removed, and the T cells were collected from the bottom chambers and analyzed using a CytoFLEX flow cytometer (Beckman Coulter). Transwell illustrations (Figure 1c) were created with BioRender.com.

(a) Immunostaining of acetylated α-tubulin (green) together with Hoechst labeled nuclei (blue) superimposed on a phase-contrast image of representative B16F10 cells pretreated with carrier (DMSO) or ZMP (50 nM) for 3 h, washed, and subsequently settled on a fibronectin-coated surface. The contours of the cell edges are outlined in white. (b) Percentage of B16F10 cells spread on fibronectin after treatment with carrier (DMSO) or ZMP (*n* = 300). Data are represented as mean ± SEM from two independent experiments. **p(0.0014). *ns *= nonsignificant. (c) Illustrations representing chemotactic and haptotactic migration of B16F10 cells, respectively, toward soluble HFG or fibronectin coated on the lower aspect of the transwell filter. (d, e) Chemotactic and haptotactic migration of B16F10 cells treated with carrier (DMSO), ZMP (50 nM for 3 h), or jasplakinolide (Jasp, 1 µM for 30 min), washed and introduced in a transwell filter (8 µm pores). Data are represented as mean ± SEM from three independent experiments. ****p < 0.0001. One-way (b) or two-way (d, e) ANOVA with Bonferroni’s post hoc test.

### Tumor cell spreading on different ECM ligands and endothelial-deposited basement membrane

Β16F10 (4 × 10^4^) pretreated with 50 nM ZMP or carrier control (DMSO, 1:2000 dilution) for 3 h were trypsinized and introduced into a μ-Slide VI0.4 ibiTreat (ibidi) previously coated with murine laminin (Corning Cat# 354232) or bovine fibronectin (Sigma-Aldrich, Cat# F1141), both at a concentration of 5 µg/ml for 1 h at RT. Alternatively the cells were allowed to spread on an endothelial-deposited ECM substrate prepared by the removal of bEnd.3 cells grown on gelatin 2 d before. The ECs detached from the substrate by a 30 min treatment with 10 mM EDTA left behind a basement membrane (ECM) deposited on the original gelatin-coated slide. Images were acquired at a rate of one frame every 10 min for 3 h using an IX83 inverted microscope (Olympus) equipped with UPlanFLN 20×/0.50 Ph1 ∞/0.17/FN 26.5 objective (Olympus). Cell spreading visualized by videomicroscopy was manually determined as described [46]. Nuclear movements inside single tumor cells spread on the different ECM ligands were tracked using Fiji (Tracking plugin), and data were subsequently plotted using the software Chemotaxis and Migration tool (ibidi).

### Light sheet fluorescent microscopy (LSFM) of tumor cells and inside lung vessels

Β16F10 (2 × 10^4^) pretreated with 50 nM ZMP or carrier control (DMSO, 1:2000 dilution) for 3 h were trypsinized and labeled with CMTMR dye (Thermo Fisher Scientific, Cat# C2927), 10 µM for 30 min according to the manufacturer’s instructions, and were injected in the retro-orbital sinus of recipient mice. Euthanasia by administration of sodium pentobarbital (200 mg/kg) was practiced 3 h later. Blood capillaries were labeled 15 min before the animal sacrifice by intravenous injection of 6 µg of an Alexa 647-conjugated anti-CD31 mAb (BioLegend, Cat# 102416, RRID:AB_493410). Immediately after the sacrifice, mice were transcardially perfused with PBS and the lungs inflated via the trachea with low gelling agarose (Sigma-Aldrich, Cat# 9045), subsequently fixed with paraformaldehyde (4% in PBS) for 2 h, dehydrated, and cleared using ethyl cinnamate as described in [47]. Cleared intact lung lobes were imaged using an Ultramicroscope II (LaVision BioTec) operated by the ImspectorPro software (LaVision BioTec). For excitation, light sheet was generated by a Superk Super-continuum white light laser [emission 460–800 nm, 1 mW/nm – 3 (NKT photonics)], followed by specific excitation filters per channel. For detection optics, microscope was equipped with a single lens configuration – 4× objective – LVBT 4X UM2-BG, with an adjustable refractive index collar set to the RI of 1.56. Images were acquired by an Andor Neo sCMOS camera (2560 × 2160, pixel size 6.5 µm × 6.5 µm, Andor). Z stacks were acquired in 3 μm steps: channel configuration for GFP and EGFP excitation 470\40 emission 525\50, for CMTMR excitation 560\40 emission 630\75, and for CD31-AF647 excitation 640\30 emission 690\50.

### Image reconstruction and analysis

Three-dimensional rendering of LSFM was performed via Imaris software (Oxford Instruments). Surfaces of CMTMR-labeled tumor cells were created using volume (comprised between 100 and 50,000 µm^3^) and intensity (max of red fluorescent channel) as defining features to unequivocally separate them from background signals. Each cell was individually segmented and its distance was measured with respect to the CD31-labeled blood vessels: intravascular, extravascular, or protruding.

LSFM illustration (Figure 3a) was created with BioRender.com.

### Tumor cell accumulation and viability inside the lung vasculature

Β16F10 (2 × 10^4^) pretreated with 50 nM ZMP or carrier control (DMSO, 1:2000 dilution) for 3 h were trypsinized and labeled with CMTMR dye 10 µM for 30 min according to the manufacturer’s instructions. The different groups of cells were injected in the retro-orbital sinus of recipient mice sacrificed 3 h later following administration of sodium pentobarbital (200 mg/kg). Immediately thereafter, mice were transcardially perfused with PBS and the lungs were extracted, minced, and incubated in RPMI-1640 containing 1.5 mg/ml collagenase type 4 (Worthington Biochemical, Cat# LS004188) and 20 µg/ml DNase I (Roche, Cat# 10104159001) at 37°C for 45 min. Lung cell suspensions were pushed through a 100 µm cell strainer and centrifuged at 0.2 × *g* or 5 min at 4°C. Red blood cells (RBCs) were subsequently lysed with an RBC lysis buffer (Sigma Aldrich, Cat# R7757). The cells were resuspended in ice-cold fluorescence-activated cell sorting (FACS) buffer (PBS with 1% BSA, 0.1% sodium azide, and 5 mM EDTA), filtered through a 70 µm strainer. Additionally, the cell suspension was resuspended in Annexin V Binding buffer (BioLegend, Cat# 422201), labeled with APC-conjugated Annexin V antibody (BioLegend, Cat# 640920) for 15 min at RT, and analyzed using a CytoFLEX flow cytometer (Beckman Coulter).

### Analysis of effector T cells migration under shear flow

HDBECs were plated at confluence on plastic culture dishes spotted with fibronectin (20 µg/ml in PBS) and, a day later, stimulated for 3 h with IL-1β (PeproTech, Cat# 200-01B) at 2 ng/ml. EC-coated plates were assembled in a flow chamber [44]. Effector T cells pretreated with 100 nM ZMP or carrier control (DMSO) for 6 h were washed and perfused over the EC monolayer in binding medium (as described above) for 40 s at a shear stress of 1.5 dyn/cm^2^ and then subjected to a shear stress of 5 dyn/cm^2^ for 10 min. Images were acquired at a rate of four frames per minute using an Olympus IX83 microscope. For analysis of migratory categories, T cells accumulated in at least three fields of view (60 cells per field) were individually tracked and categorized as described [44].

### Statistical analysis

Data in graphs are represented as mean ± standard error of the mean (SEM). Student’s two-tailed unpaired *t* test was used to determine the significance of the difference between means of two groups. One or two-way analysis of variance (ANOVA) tests were used to compare means among three or more independent groups. Significance was set to *p* < 0.05. Statistical details of experiments can be also found in the figure legends.

## Results and discussion

### MT disassembly is critical for melanoma chemotaxis and haptotaxis but not for adhesion and spreading

To assess how interference with MT turnover in Β16F10 cells affects their adhesion, spreading, and directed migration properties, we exposed these cells to ZMP for short periods of time. The treatment of tumor cells with ZMP (50 nM) for 3 h following removal of the drug by cell washing did not affect Β16F10 viability but altered MT distribution and bundling as depicted by live imaging of fluorescent α-tubulin (Video 1). Furthermore, ZMP-treated cells contained dramatically elevated levels of stable MTs, marked by α-tubulin acetylation (Figure 1a). Notably, during Β16F10 spreading on fibronectin, ZMP-stabilized acetylated MTs were enriched in both anterior and posterior compartments around the tumor cell nucleus in contrast to their localization in a small region in front of the nucleus of untreated cells (Figure 1a). Interestingly, however, the ZMP treatment did not prevent Β16F10 adhesion to and spreading on fibronectin, as opposed to jasplakinolide (Jasp), a potent inhibitor of actin disassembly [48,49] (Figure 1b). This result suggested that interference with MT turnover does not inhibit Β16F10 spreading on ECM ligands, whereas interference with actin turnover by jasplakinolide treatment does. Furthermore, ZMP treatment also did not interfere with B16F10 spreading on the ECM glycoprotein laminin, a well-studied adhesive ligand for B16 melanoma [50] (Suppl. Figure 1a), nor with the random displacement of their nuclei during this spreading (Suppl. Figure 1b, Video 2). Collectively, these results suggested that interference with MT turnover in Β16 melanoma cells does not inhibit their adhesion, spreading, or random nuclear motility.

To assess MT involvement in directional Β16F10 motility driven by chemotactic gradients or by adhesive gradients, a process referred to as haptotaxis [51] (Figure 1c), we next determined if ZMP-treated Β16F10 cells can migrate toward a gradient of soluble HGF, a potent chemoattractant for these melanoma cells (Suppl. Figure 2). Notably, ZMP-treated cells failed to cross large pore transwells in response to an HGF gradient (Figure 1d). Furthermore, Β16F10 haptotaxis toward fibronectin, coated on the lower aspects of similar transwell filters, was also impaired by ZMP treatment (Figure 1e). As expected, jasplakinolide treatment abolished both chemotactic and haptotactic melanoma migration through large pore transwells (Figure 1d, e). Since Β16 chemotaxis and haptotaxis were both sensitive to ZMP treatment, we conclude that MT turnover in these cancer cells is essential for directional cancer cell motility toward different types of cues. The dynamic interaction of the nucleus with the actomyosin cytoskeleton is required to ensure proper nuclear deformation to successfully squeeze the cell through small but not through large constrictions [52,53]. We have recently shown that nucleus deformation is the rate-limiting step for Β16F10 to pass through constrictions smaller than the nucleus [39]. Since ZMP-treated Β16F10 cells failed to cross large pores, these results suggest that interference with MT disassembly blocks the chemotaxis and haptotaxis of these cells at a distinct step, possibly prior to actomyosin-driven nuclear deformation.

**Figure 2.**
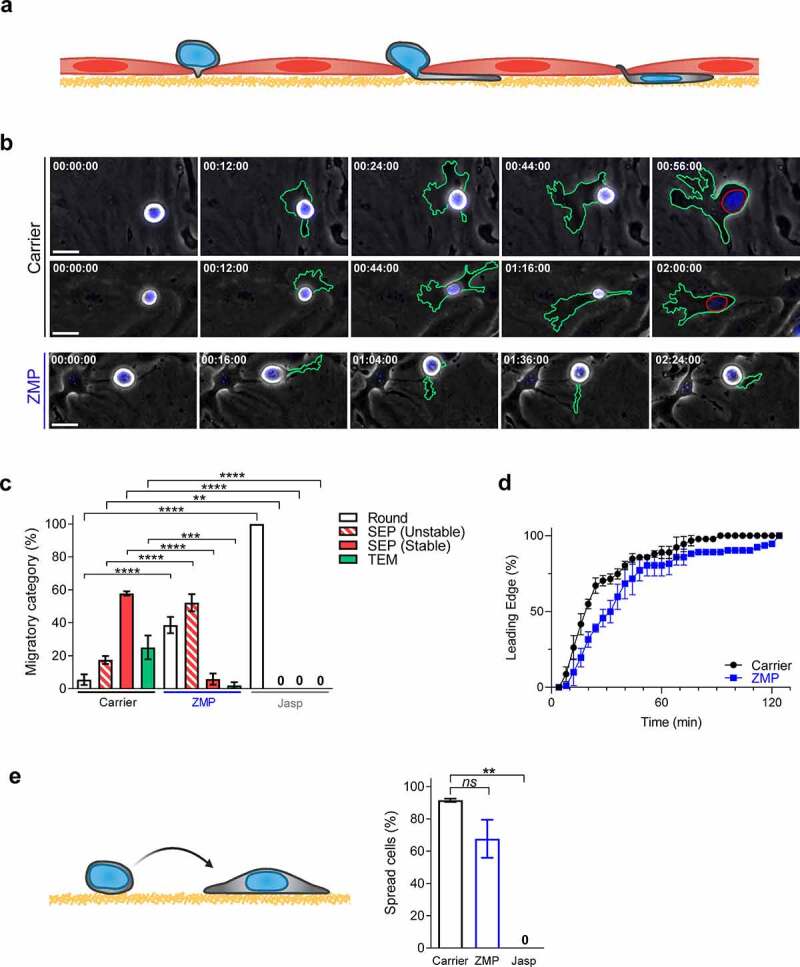
Tubulin turnover is critical for B16F10 transendothelial migration but not for spreading on ECM

(a) Illustration depicting a B16F10 cell (gray) undergoing TEM in between endothelial cells (light red). Basement membrane (deposited ECM) is represented in yellow. (b) Serial images of representative B16F10 cells pretreated with DMSO or ZMP and labeled with Hoechst 33342, interacting with bEnd.3 endothelial monolayers. Time intervals of Videos 3–5 are depicted in each image. The contours of the melanoma cells and their nuclei are outlined in each image in green and red, respectively. Scale bars, 20 µm. (c) Migratory categories of B16F10 cells pretreated with carrier (DMSO), ZMP (50 nM for 3 h), or Jasp (1 µM for 30 min), interacting with unstimulated bEnd.3 cells (*n* = 60). Data are represented as mean ± SEM of three independent experiments. **p(Carrier vs Jasp) = 0.0025; ***p(Carrier vs ZMP) = 0.0001; ****p < 0.0001. (d) Kinetics of insertion of the B16F10’s leading edge underneath a bEnd.3 endothelial monolayer (*n* = 90). Data are represented as mean ± SEM of three independent experiments. (e) Percentage of B16F10 cells spreading on bEnd.3-derived basement membrane (ECM) after pretreatment with carrier (DMSO), ZMP, or Jasp (*n* = 100). Cells were settled on the ECM-coated substrate for 2 h (left scheme). Data are represented as mean ± SEM from two independent experiments. **p(Carrier vs Jasp) = 0.0050; *ns = *nonsignificant. Two-way (c) or one-way (e) ANOVA with Bonferroni’s post hoc test.

### MT turnover is critical for cancer cell TEM

Cancer cell TEM in vitro involves sequential steps (Figure 2a): spreading over the EC surface, protrusion through paracellular EC junctions, generation of a stable subendothelial pseudopodia that displaces the EC attachments to the endothelial-deposited ECM (basement membrane) and nucleus squeezing below the endothelial monolayer [39] (Figure 2b and Videos 3 and 4). As expected, interference with actin turnover using Jasp blocked tumor TEM by abolishing the ability of Β16F10 cells to initially spread over the EC monolayer (Figure 2c). ZMP treatment also reduced Β16F10 melanoma spreading on the endothelial monolayer (Figure 2c, round category). Interestingly, however, ZMP-treated cells could readily protrude through endothelial junctions, with small protrusions extended underneath the EC monolayer (Figure 2b–d). Interestingly, the rate of Β16F10 protrusion into the EC junctions was only slightly delayed by ZMP pretreatment (Figure 2d), suggesting that inhibiting MT disassembly does not interfere with the ability of these melanoma cells to sense chemotactic cues across the endothelial monolayer. Furthermore, when settled on the endothelial derived basement membrane, a deposition of ECM [54] ZMP-treated cells readily spread on this substrate (Figure 2e). Nevertheless, the ZMP treatment reduced the stability of the subendothelial pseudopodia generated by Β16F10 protrusions underneath the endothelium during active TEM (Figure 2b, c and Video 5). Consequently, the ZMP-stabilized MTs prevented B16F10 cells from squeezing their nuclei through endothelial junctions and into their subendothelial pseudopodia (Figure 2b, c and Video 5). Collectively, these experiments indicate that an MT disassembly step is essential for the ability of B16 cells to generate productive subendothelial pseudopodia followed by nuclear squeezing through endothelial junctions, critical checkpoints of successful TEM. This disassembly is likely required to release sequestered Rho GEFs and activate myosin machineries [2] presumably critical for both pseudopodia stability and nuclear translocation underlying productive melanoma TEM.

Β16F10 cells express high surface levels of β1 and β3 integrin subfamilies (Suppl. Figure 3a). Surprisingly, although the ability of these cells to optimally spread on the subendothelial basement membrane was blocked by functional blocking mAbs to both integrin subfamilies (Suppl. Figure 3b), normal Β16F10 TEM took place in the presence of saturating levels of these blocking mAbs (Suppl. Figure 3c). Thus, although Β16 cells can use their integrins to directly spread on the endothelial basement membrane deposited underneath the endothelial monolayer, the ability of these tumor cells to protrude underneath the monolayer, establish stable subendothelial pseudopodia on this ECM layer during TEM, and squeeze their nuclei does not require functional tumor β1 and β3 integrins. Taken together, these results suggest that in our experimental setting Β16F10 cells use integrin-independent cues for crossing endothelial monolayers.

**Figure 3.**
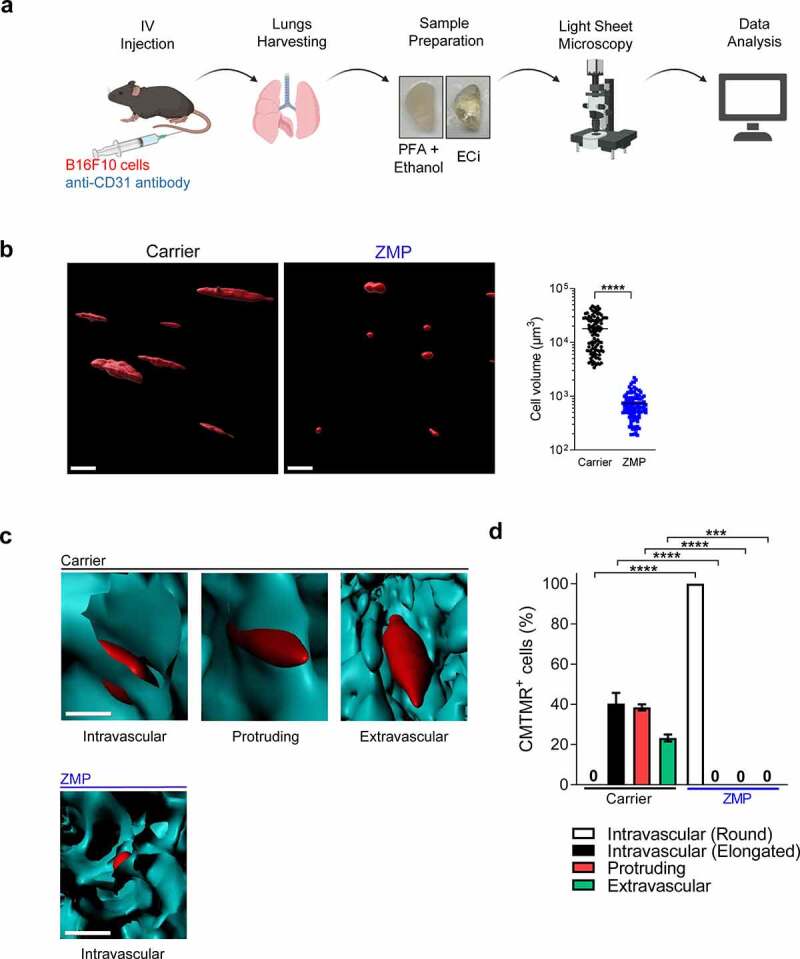
ZMP-treated B16F10 cells fail to spread and cross lung capillaries in vivo

(a) Schematic representation of the LSFM analysis. (b) 3D visualization of B16F10 cells (red) present in the lungs 3 h after IV injection. B16F10 cells were pretreated with carrier (DMSO) or ZMP (50 nM) for 3 h, labeled with CMTMR, and injected into recipient mice. Scale bars, 100 μm. In the right panel, cell volume was quantified using Imaris software (*n* = 100). See also Video 6. (c) Representative 3D images of intravascular, extravascular, and protrusive tumor cells within CD31-labeled blood vessels (cyan). Scale bar, 100 µm. See also Videos 7–10. (d) Fractions of cells pretreated with carrier (DMSO) or ZMP, injected as outlined in (b) and present in a volume of 5 × 10^9^ µm^3^ of the left lung lobe (*n* = 40). Data are represented as mean ± SEM of two independent experiments. ***p(0.0002); ****p < 0.0001. Student’s two-tailed unpaired *t* test (b) or two-way ANOVA with Bonferroni’s post hoc test (d).

### MT disassembly in Β16F10 cells is critical for elongation and crossing of lung capillaries in vivo

We next assessed how ZMP treatment in vitro affects Β16F10 migration through lung capillaries in vivo (Figure 3a). Cells were identically pretreated with ZMP or control medium as in previous sections, labeled with the cell tracker CMTMR and intravenously (IV) injected into recipient mice. IV-injected Β16F10 cells reached the lung vasculature within minutes. While most of the control cells present in the lungs were elongated, nearly all ZMP-treated cells were depicted as rounded with much lower volumes (Figure 3b and Video 6). High-resolution analysis of blood vessels stained with CD31-specific mAb indicated that at the selected time, i.e., 3 h after IV injection of control CMTMR-labeled B16F10 cells, a fraction of these cells had extravasated across the lung blood vessels or were in the process of emigration (Figure 3c, d and Videos 7–9). In contrast, all ZMP-treated B16F10 cells remained entrapped inside the pulmonary blood vessels (Figure 3c and Video 10). Importantly, the viability of ZMP- and control-treated Β16F10 cells recovered from total lung suspensions was high and comparable, ruling out that the inability of ZMP cells to elongate inside lung capillaries was due to accelerated cell death (data not shown). Collectively these results further suggest that intact MTs are critical for the ability of Β16F10 cells reaching the lung capillaries to normally spread, elongate, and extravasate through these blood vessels.

### MT disassembly in T cells is not obligatory for chemotactic squeezing or TEM across endothelial monolayers

**Figure 4.**
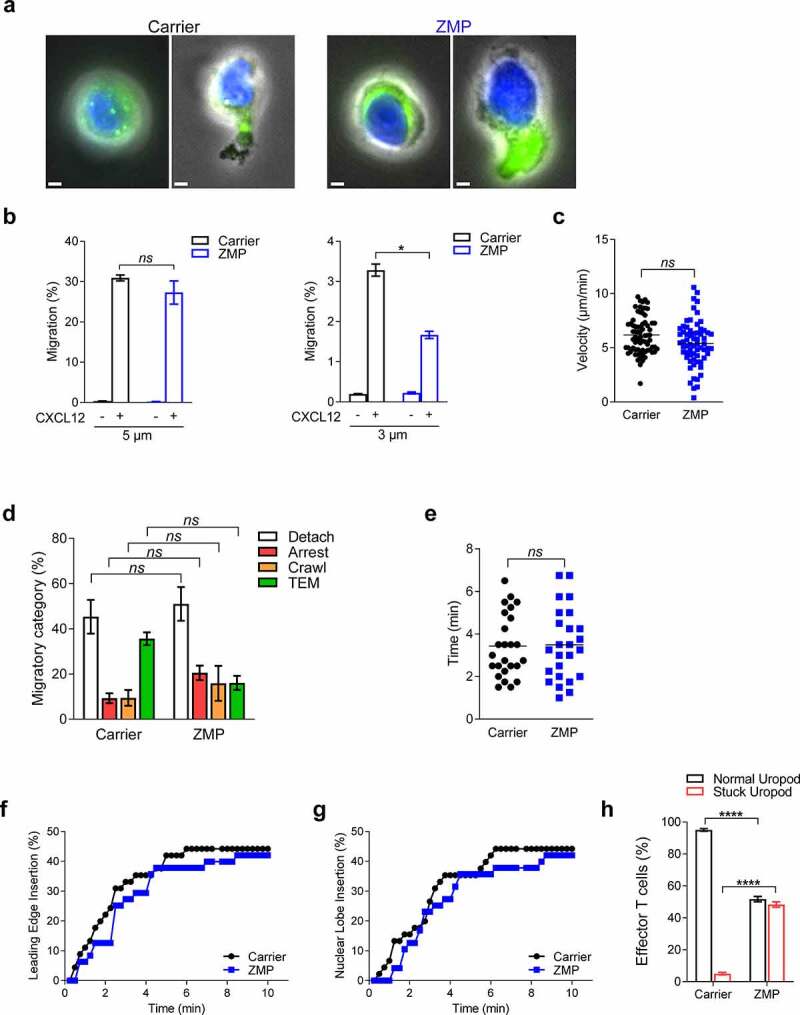
ZMP pretreatment does not affect T cell chemotaxis and nuclear translocation during TEM but slows uropod release

Since MTs in fast-moving cells like T cells are enriched behind the nucleus [15,18], we finally tested the effects of ZMP treatment on either random or directional migration of effector T cells. As expected, ZMP-treated T cells contained higher content of acetylated tubulin, enriched behind their nuclei (Figure 4a). In sharp contrast to Β16 cells, the chemotaxis of ZMP-treated T cells across large rigid pores, i.e., transwell filters, remained normal and even under extreme confinements these T cells could still squeeze in response to a chemotactic cue (Figure 4b). ZMP-treated T cells also normally locomoted on a 2D surface coated with a chemokine carpet (Figure 4c). The ability of ZMP-treated effector T cells to spread on endothelial monolayers, protrude through these monolayers, and stabilize subendothelial pseudopodia, processes mediated by signals from endothelial presented chemokines [40,55,56], was also intact (Figure 4d–f). Furthermore, the ability of ZMP-treated T cells to rapidly squeeze their nuclei during active TEM was also unaltered, as indicated by the kinetics of nuclear squeezing through endothelial junctions and the overall kinetics of T cell TEM (Figure 4d, g). Nevertheless, a portion of ZMP-treated T cells that successfully squeezed their nuclei underneath the endothelium failed to release their uropod from the apical endothelial aspect (Figure 4h). This specific TEM defect of ZMP-treated T cells did not resemble, however, those of myosin IIa-deficient T cells [57]. MyoIIA is the major contractility-promoting myosin in T cells and accumulates at the rear of T cells undergoing TEM [57]. Murine T cells deficient in this myosin extend pseudopodia and project a substantial portion of their cytoplasm but fail to squeeze their nuclei through the endothelial wall [57]. Our results therefore highlight a specialized role of MT disassembly in T cell TEM, namely the regulation of the uropod contractility rather than of an earlier myosin-driven nuclear translocation into the T cell leading edge (pseudopodium). This specialized role is consistent with the location of the MTOC in locomoting T cells, i.e., right behind the nucleus and in front of the lymphocyte’s uropod. Notably, the T cell MTOC can be translocated to the front of the nucleus when T cells, similar to other leukocytes, generate immune synapses, in which the MTOC is involved in coordinating directed secretion of cytokines as well as of cytotoxic and lytic granules into the T cell contact with its target cell [58–61]. In phagocytic leukocytes, MTs also serve as tracks for vesicular traffic in both phagosome formation and phagosome maturation [62]. It is therefore likely that ZMP-treated T cells while largely maintaining their directional migration and barrier crossing capacities are severely deficient in their ability to generate functional immune synapses with their cellular targets.

(a) Representative immunostaining of acetylated α-tubulin (green) together with Hoechst-labeled nuclei (blue) superimposed on a phase-contrast image of human effector T cells treated with carrier (DMSO) or ZMP (100 nM) for 6 h and subsequently settled on poly-L-lysine and CXCL12-coated surface. Scale bars, 2 µm. (b) Chemotactic migration of effector T cells toward CXCL12 (100 ng/ml). T cells were pretreated with carrier (DMSO) or ZMP (100 nM) for 6 h, washed, and introduced in transwell filters with the indicated pore sizes. Data are represented as mean ± SEM from two independent experiments. *p(0.0116); *ns = *nonsignificant. (c) Velocity of individual human effector T cells pretreated with carrier (DMSO) or ZMP (100 nM) for 6 h, randomly locomoting on a CXCL12-coated surface (*n* = 71, carrier; *n* = 65, ZMP). Data are represented as mean ± SEM from two independent experiments. (d) Migratory categories of carrier (DMSO) or ZMP-pretreated human effector T cells interacting with IL‐1β-prestimulated human endothelial cells under shear flow (*n* = 400). Arrested T cells were subcategorized into cells that arrested without subsequent protrusion and pseudopodia formation vs. cells that squeezed their nucleus through the monolayer but had their uropod stuck on the apical endothelial surface. Data are represented as mean ± SEM of two independent experiments. The adhesive and migratory categories are further described in the Materials and methods section. (e) Overall TEM duration (minutes) of individual effector T cells across inflamed endothelial monolayers (*n* = 25). Data are represented as mean ± SEM from two independent experiments. (f) Fraction of effector T cells that projected a subendothelial leading edge underneath the monolayer at the indicated time points. (g) Fraction of effector T cells that inserted their nuclei underneath the endothelial monolayer at the indicated time points. (h) T cells with normal or stuck uropod during TEM (*n* = 100). ****p < 0.0001. Student’s two-tailed unpaired *t* test (b, c, e, h) or two-way ANOVA with Bonferroni’s post hoc test (d).

In conclusion, our results suggest that MT disassembly plays distinct roles in Β16 melanoma and in T cell TEM, reflecting the distinct distribution of their MTOC in mesenchymal-derived slow-moving cells like melanoma cells versus highly motile leukocytes like T cells (Suppl. Figure 4). It is likely that in both types of cells, MT disassembly plays a role in releasing various MT sequestered Rho GEFs. Nevertheless, in melanoma cells and possibly in other solid tumors, MT assemblies generated in front of the cell nuclei impose restrictions on these large and stiff organelles restraining them from translocating into the leading edge during TEM and also during directional cell migration through rigid confinements. This specific role of MT disassembly does not seem to involve abnormal integrin focal adhesions since ZMP-treated melanoma cells extended normal protrusions during integrin-mediated spreading on endothelial-deposited ECM. Further extension of the present work to other types of cancer cells, migratory ECs, and motile fibroblasts may help substantiate this conclusion. Such extension will also shed new light on the regulation of this key checkpoint by MT deacetylases [23], as well as by specific MT and actomyosin-binding partners, particularly motor proteins and MAPs [25,26]. Identification of these effector molecules and their mode of activity may also aid in the design of new selective inhibitors of cancer cell migration and cancer metastasis.

## Supplementary Material

Supplemental Material
